# P01-035 – Long-term IV colchicine in oral colchicines failure

**DOI:** 10.1186/1546-0096-11-S1-A39

**Published:** 2013-11-08

**Authors:** O Feld, I Ben Zvi, OL Kukuy, A Livneh

**Affiliations:** 1 Medicine F, SHEBA MEDICAL CENTER, Rammat Gan, Israel; 2 Nephrology and Hypertension Institute, Sheba medical center, Rammat Gan, Israel; 3 Sackler Faculty of Medicine, Tel Aviv University, Tel Aviv, Israel; 4 Heller Institute for Medical Research, Sheba medical center, Rammat Gan, Israel

## Introduction

Various levels of oral colchicine resistance is still an unmet challenge of FMF treatment. IV colchicine was shown to be effective and safe answer, but its long term outcomes are not known.

## Objectives

To determine long term effectiveness and safety of IV colchicine treatment in oral colchicine refractory or intolarant FMF patients.

## Methods

Included were all patients, experiencing ≥1 attack per month, or intolerant to adequate dose of oral colchicin, for which they receive IV colchicine (pharmacy preparation under government control) for at least 1 year. Retrieval of data was based on patient interviews, files and a detailed questionnaire, focusing on clinical, demographic and genetic data. Effect of colchicine was determined, by computing attack rate, duration and intensity, the later with 1-10 scale.

## Results

Ten of 11 identified patients, on long term IV colchicine treatment, consented to partake. Treatment lasted 3.6 ±2.7 (1- 10) years. More than 50% reduction in the rate of abdominal, chest, joint and skin attacks was noted by 8 of 10 patients. The intensity of the attacks dropped by a mean of 50±22% and the duration by 40±20%. In 4 patients the favorable effect has decreased partially, but in only one treatment was stopped for this reason. Treatment was terminated in another 2 for loss of venous access (1) and for paresthesis. Adverse effects included diarrhea (1 patient), vomiting (2), injection site pain (3), headache (1), muscle pain (1), injection site phlebitis (1) and arm paresthesis (1).

## Conclusion

Long term parenteral colchicine treatment proved effective and safe. Downloading I.V. colchicine off the shelves due to intoxication associated with uncontrolled and unjustified use for back pain, had serious negative impact on our armamentarium for oral colchicine nonresponsiveness and intolerance.

## Disclosure of interest

None declared.

